# Radiologic–Pathologic Discordance After Image-Guided Breast Biopsy: A Systematic Review of Prevalence and Outcomes

**DOI:** 10.3390/medsci14020229

**Published:** 2026-04-30

**Authors:** Pirada Yincharoen, Crystal Pravina Sharma, Weeratian Tawanwongsri

**Affiliations:** 1 Department of Surgery, School of Medicine, Walailak University, Nakhon Si Thammarat 80160, Thailand; 2 Surgical Intensive Care Unit, University Hospital Dresden, 01307 Dresden, Germany; 3 Division of Dermatology, Department of Internal Medicine, School of Medicine, Walailak University, Nakhon Si Thammarat 80160, Thailand

**Keywords:** breast neoplasms, biopsy, needle, image-guided biopsy, diagnostic errors, false-negative reactions

## Abstract

Background: Radiologic–pathologic discordance remains an important concern owing to the absence of standardized guidelines. This systematic review aimed to summarize the prevalence of discordant benign outcomes, defined as suspicious imaging findings with benign biopsy histology insufficient to explain the imaging abnormality, and to quantify malignancy upgrades subsequent to additional tissue assessment. Methods: This review was conducted in accordance with PRISMA 2020 guidelines and was prospectively registered. Eligible studies reported primary patient-level or aggregated data on radiologic–pathologic correlations post-image-guided breast biopsy and provided extractable data on discordant benign prevalence and/or subsequent malignancy upgrades. Results: Twenty-three studies were included. Lesion-/biopsy-based cohorts focused on biopsied abnormalities for analysis. Twelve studies directly estimated discordant-benign prevalence, whereas 11 studies did not, as study designs were discrepant-only, lesion-defined, or excision-restricted. Unselected cohorts with a cohort-wide correlation reported 1.2–5.3% discordant benign prevalence for all biopsies. When restricted to excised lesions, the discordant benign ascertainment rate was 7.4%, representing an excision-ascertained subset rather than the cohort-wide prevalence. Using benign-biopsy denominators, the discordance rate was 1.5–19.2%. Malignancy upgrades among discordant benign lesions ranged from 0 to 100% in selected subsets; however, several clinically relevant cohorts reported representative rates of approximately 20–40%, with some high-risk cohorts exceeding 50%. Conclusions: Discordant benign biopsy results are rare in unselected biopsy populations but carry a clinically meaningful upgrade risk, which warrants structured radiologic–pathologic correlation and prompt diagnostic resolution through repeat sampling or excision. Improvements in comparability and management algorithms require standardized definitions, uniform denominators aligned with all biopsied lesions, and prospective multicenter designs.

## 1. Introduction

Breast cancer is a major global health issue and the most commonly diagnosed cancer among women worldwide [1,2]. An estimated 2.089 million women received new diagnoses, and more than 626,000 deaths were reported, highlighting the impact of breast cancer mortality on women [2]. Although incidence is highest in high-income countries, the prevalence and incidence of breast cancer are increasing in low- and middle-income countries owing to longer lifespans and westernized lifestyle changes. Developing regions face higher mortality rates owing to limited prevention, insufficient screening resources, and delayed access to treatment [2,3]. Early detection relies on standardized imaging assessments such as BI-RADS categories that guide biopsies for suspicious lesions, followed by histopathological confirmation [4,5]. However, differences in imaging sensitivity and specificity can create inconsistencies between radiologic suspicion and tissue diagnosis, affecting management and outcomes [5,6,7]. Radiologic–pathologic mismatch occurs because of (1) sampling errors, where biopsies miss key abnormalities or samples of benign tissue are obtained; (2) lesion heterogeneity, where benign parts are sampled but malignant areas are missed; and (3) interpretive limitations, including benign conditions mimicking cancer and suboptimal tissue cores influenced by technical and operator factors [8,9,10,11,12,13].

Radiologic–pathologic discordance occurs when histopathologic results from image-guided biopsies do not match the imaging findings [14]. For the purposes of this review, discordant benign was operationally defined as suspicious imaging findings (typically BI-RADS 4–5 or equivalent clinical concern) with benign or otherwise non-malignant histopathology that was considered insufficient to explain the imaging abnormality after radiologic–pathologic correlation [15,16,17]. Discordance includes radiologically benign findings with malignant histology and radiologically suspicious findings with benign histology. The second scenario, called discordant benign, involves BI-RADS 4–5 abnormalities that yield a benign pathology. However, these results do not explain the imaging findings, suggesting sampling errors or interpretation issues. Additional tissue samples obtained by repeat biopsy or surgical excision are required to reduce the risk of missed malignancies [8,12,18,19]. Radiologically benign lesions with malignant histology are less common. Some cancers may show unusual imaging features or require biopsy owing to symptoms, clinician concerns, or changes over time, rather than suspicious imaging patterns [20,21,22]. Managing discordance requires structured radiologic–pathologic correlation and multidisciplinary review. This issue has been addressed at inter-departmental conferences to ensure accurate sampling and diagnostic validity. Further sampling or excision is recommended when benign diagnoses fail to explain the imaging findings [9,23]. Recent studies have highlighted the importance of discordant benign lesions. Wan et al. found a 2.1% rate of discordant benign lesions in 3080 biopsies [19]. Malignancy was confirmed in many cases with further evaluation. Malignancy rates reported after investigating discordant benign lesions were 55% [18], 26% [8], and 30.9% [19]. Discordant benign results are important because they may represent false-negative biopsies, resulting in delayed cancer diagnosis and stage progression [9,24]. They require additional tissue sampling, increasing procedural morbidity, patient anxiety, and healthcare utilization [18,19,25].

Despite fragmented evidence across heterogeneous single-center cohorts with varying biopsy techniques, imaging modalities, and definitions of discordance, the quantification of the prevalence of radiologic–pathologic discordance after image-guided breast biopsy is needed. This systematic review aimed to determine the prevalence of radiologic–pathologic discordance following breast biopsy and to summarize clinical outcomes, including malignancy rates and management actions, after the identification of discordance.

## 2. Materials and Methods

This systematic review was conducted in accordance with the Preferred Reporting Items for Systematic Reviews and Meta-Analyses (PRISMA) 2020 guidelines (Supplementary File S1) [26]. The review protocol was prospectively registered on 17 January 2026, with the International Platform of Registered Systematic Review and Meta-Analysis Protocols (INPLASY; registration number: INPLASY202610057; DOI: 10.37766/inplasy2026.1.0057) to ensure methodological transparency and minimize the risk of selective reporting. This study was approved by the Walailak University Ethics Committee (WUEC) on WUEC-26-079-01.

### 2.1. Eligibility Criteria

Studies reporting primary patient-level or aggregated data on radiologic–pathologic correlations after image-guided breast biopsy were considered eligible. The inclusion criteria were randomized controlled trials, non-randomized comparative studies, prospective or retrospective cohort studies, and cross-sectional studies that provided extractable data on the prevalence of radiologic–pathologic discordance and/or subsequent clinical outcomes. Eligible studies were required to be published as full-text articles in English and to report explicit radiologic–pathologic correlations with lesions clearly classified as concordant or discordant (including discordant benign results). The review included human participants of any sex and adult age range (or mixed-age cohorts in which adult data were predominant or inseparable) without restrictions on ethnicity or geographic region. Imaging-detected breast lesions of any type (e.g., masses, architectural distortion, or calcifications) and any BI-RADS category were eligible if a biopsy was performed using an image-guided technique (e.g., ultrasound-guided, stereotactic, digital breast tomosynthesis-guided, or magnetic resonance imaging [MRI]-guided biopsy).

The exclusion criteria were as follows: (1) secondary research, including narrative reviews, systematic reviews, and meta-analyses; (2) editorials, expert opinions, letters, conference abstracts, theses, preprints, or technical notes lacking sufficient extractable data to estimate discordance prevalence and/or related clinical or management outcomes; (3) studies with no explicit radiologic–pathologic correlation or without a clear definition of concordant versus discordant findings; (4) non-image-guided breast biopsy studies; (5) animal or in vitro studies; (6) duplicate publications or overlapping cohorts (in which case the most comprehensive or most recent report was retained); and (7) articles addressing non-breast lesions or other organ systems, or elastolytic or pathologic entities unrelated to radiologic–pathologic discordance in breast biopsy.

For this review, radiologic–pathologic discordance was operationally defined as a benign or otherwise non-malignant histopathologic result considered insufficient to explain suspicious imaging findings after radiologic–pathologic correlation. In most included studies, suspicious imaging findings corresponded to BI-RADS 4–5 lesions or equivalent clinical concern. Cases meeting this framework were categorized as discordant benign when further tissue sampling, surgical excision, multidisciplinary review, or close imaging follow-up was considered necessary. Because terminology and denominators varied across studies, study-specific labels (e.g., discordant benign, discrepant benign findings, imaging–histologic mismatch) were harmonized to this predefined review-level framework during data extraction and synthesis.

### 2.2. Information Sources and Search Strategy

A comprehensive and systematic literature search was conducted in MEDLINE (via PubMed), Scopus, and the Directory of Open Access Journals (DOAJ) from database inception to 17 January 2026. The search was restricted to publications in English. Secondary studies, including narrative reviews, systematic reviews, and meta-analyses, were excluded from the database search. In addition, we screened the reference lists of all included studies and relevant reviews and conducted backward and forward citation tracking (citation chasing), to identify additional eligible primary studies not captured by the electronic search. Corresponding authors were contacted when necessary to clarify unclear information or request missing data relevant to radiologic–pathologic discordance or downstream clinical outcomes.

Electronic search strategies were developed a priori and tailored to each database using a combination of controlled vocabulary terms (where applicable) and free-text keywords related to breast biopsy, image-guided techniques, and radiologic–pathologic discordance. The complete search strategy, including the applied filters and limits, is as follows.

Scopus:

TITLE-ABS-KEY ((breast OR mammary) AND (biops* OR CNB OR VAB OR “core needle” OR “vacuum assisted”) AND ((radiolog* W/3 patholog*) OR “radiology-pathology” OR “radiologic-pathologic” OR “imaging-pathology”) AND (discord* OR discrepanc* OR concord*)) AND NOT TITLE-ABS-KEY (thyroid OR prostate OR lung OR liver OR kidney OR cervical OR “lymph node”) AND (LIMIT-TO (DOCTYPE, “ar”)) AND (LIMIT-TO (LANGUAGE, “English”))

MEDLINE (via PubMed):

((“Breast”[Mesh] OR breast[tiab] OR mammary[tiab]) AND (“Biopsy, Needle”[Mesh] OR biopsy[tiab] OR “core needle”[tiab] OR CNB[tiab] OR “vacuum assisted”[tiab] OR VAB[tiab]) AND (“radiology pathology”[tiab] OR “radiologic-pathologic”[tiab] OR “radiology-pathology”[tiab] OR “imaging-pathology”[tiab] OR (radiolog*[tiab] AND patholog*[tiab])) AND (discordan*[tiab] OR discrepanc*[tiab] OR “discordant benign”[tiab]) AND (excision[tiab] OR excisional[tiab] OR “repeat biopsy”[tiab] OR rebiops*[tiab] OR upgrade*[tiab] OR malignan*[tiab] OR “false negative”[tiab] OR underestimat*[tiab])) AND english[lang] AND humans[Mesh] NOT (review[pt] OR editorial[pt] OR letter[pt] OR comment[pt])

Directory of Open Access Journals (DOAJ):

breast biopsy “radiology-pathology” discordance

The final search results were exported to EndNote X9 (Clarivate Analytics, Philadelphia, PA, USA), and duplicates were removed prior to screening and selection.

### 2.3. Selection Process

All records retrieved from electronic database searches were collated, and duplicate citations were identified and manually removed. Two reviewers independently screened the titles and abstracts to assess potential eligibility, followed by full-text evaluation of the articles deemed relevant. Any disagreements between reviewers at any stage were resolved through discussion and consensus. Automated tools, machine learning algorithms, or artificial intelligence–assisted screening software were not used at any stage of the study selection process.

### 2.4. Data Extraction and Synthesis

A standardized data extraction form was developed a priori and piloted on a subset of the included studies to ensure consistency and completeness. Two reviewers independently extracted data from each eligible article, and any discrepancies were resolved through discussion between the reviewers. No automated tools or artificial intelligence–assisted systems were used for the data extraction. A detailed study-level extraction dataset containing key variables (e.g., BI-RADS categories, biopsy technique, follow-up, denominator framework, management pathway, and upgrade outcomes) was prepared and is provided as Supplementary File S2.

The following data were extracted: (1) study characteristics, including first author, year of publication, country, study design, setting (single-center or multicenter), sample size, and duration of follow-up; (2) participant characteristics, including age, sex, and relevant clinical information when reported; (3) imaging characteristics, including imaging modality (e.g., ultrasound, stereotactic, digital breast tomosynthesis, or MRI), lesion type (mass, calcifications, architectural distortion, or other), and BI-RADS category; (4) biopsy-related details, including biopsy technique (core needle biopsy, vacuum-assisted biopsy [VAB]), needle gauge, and guidance modality; (5) radiologic–pathologic correlation outcomes, including classification as concordant or discordant, with specific identification of discordant benign results; and (6) downstream clinical outcomes following discordance, including repeat biopsy, VAB, surgical excision, imaging surveillance, and malignancy (upgrade) rates, as well as the histopathologic nature of any upgraded lesions (e.g., ductal carcinoma in situ [DCIS] or invasive carcinoma), when available. Additional extracted variables included factors potentially associated with discordance or malignancy upgrade (e.g., lesion size, imaging features, and biopsy method), as well as reported funding sources and conflicts of interest. Items not reported in the original publications were recorded as “not reported,” and no data were imputed. To improve comparability across heterogeneous studies, reported discordance categories were mapped to the predefined review-level operational framework. This approach was used to minimize definitional heterogeneity and permit more consistent synthesis across studies with differing terminology or classification methods. When sufficient detail was available, outcomes were additionally stratified according to denominator type (all biopsies, benign biopsies, or excision-restricted cohorts).

These findings were primarily synthesized using structured narrative synthesis and tabulation. For reproducibility, studies were grouped a priori according to denominator framework and design characteristics, including (1) all-biopsy cohorts, (2) benign-biopsy cohorts, (3) excision-restricted cohorts, (4) lesion-defined selected cohorts, and (5) high-suspicion subgroups. Outcomes were then summarized within these strata before cross-study comparison. Greater interpretive emphasis was placed on studies with cohort-wide ascertainment, explicit denominators, and directly calculable prevalence estimates, whereas findings from highly selected or verification-restricted cohorts were interpreted as exploratory or context-specific. When numerical pooling was inappropriate, consistency of direction, magnitude ranges, and recurring clinical patterns were narratively assessed across studies. Categorical variables were summarized as counts and proportions with explicit denominators, whereas continuous variables were reported as means (standard deviations) or medians (interquartile ranges), as provided in the original studies. A meta-analysis was not performed because substantial clinical and methodological heterogeneity across studies was judged to preclude reliable pooling. Key sources of heterogeneity included variable definitions of discordance, inconsistent denominator frameworks, differences in imaging modality and biopsy technique, selected versus unselected study populations, and nonuniform outcome verification or follow-up pathways. Under these conditions, pooled estimates were considered potentially misleading. If sufficiently homogeneous data were available for a given outcome, pooled estimates (e.g., prevalence, risk ratios, odds ratios, or mean differences) using a random-effects model were considered, with statistical heterogeneity assessed using the *I*^2^ statistic and Cochran’s Q test. The exploration of heterogeneity was descriptive and based on clinically relevant subgroups such as the imaging modality, biopsy technique, and lesion type. Sensitivity analyses, where feasible, repeated the key summaries after excluding studies with a high risk of bias.

### 2.5. Risk of Bias Assessment

Two reviewers independently assessed the methodological quality and risk of bias using the Joanna Briggs Institute (JBI) Critical Appraisal tools [27,28,29]. The JBI Critical Appraisal Checklist for Studies Reporting Prevalence Data was used for radiologic–pathologic discordance studies, whereas the Checklist for Cohort Studies was used for studies evaluating downstream clinical outcomes. Discrepancies were resolved through discussion. No automated tools were used to assess risk of bias. The results were summarized and considered when interpreting the evidence certainty. Given the anticipated observational and non-comparative nature of the studies, formal statistical methods for assessing reporting bias were deemed inappropriate. To minimize bias from incomplete reporting, outcomes were comprehensively extracted from each study and compared with prespecified methods when feasible, and selective reporting was documented. When important data were missing, the corresponding authors were contacted. We did not apply GRADE because the evidence was observational and highly heterogeneous (definitions, denominators, and outcome verification pathways), which made it difficult to interpret a single certainty rating. Instead, we assessed the study-level risk of bias and qualitatively discussed the evidence limitations.

## 3. Results

**Figure 1:**
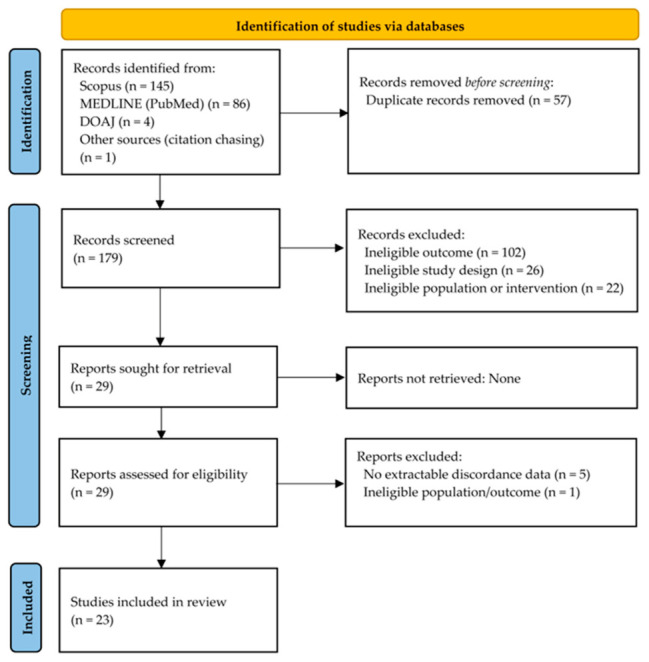
PRISMA flow diagram of the study selection process. DOAJ, Directory of Open Access Journals; MEDLINE, Medical Literature Analysis and Retrieval System Online; PRISMA, Preferred Reporting Items for Systematic Reviews and Meta-Analyses.

A total of 23 studies were included in the qualitative analysis (Figure 1). Table 1 summarizes the study characteristics, including setting/design and period, biopsy and imaging context, and radiologic–pathologic correlation workflows (including discordance adjudication and downstream management). Participant characteristics were variably reported; 15 of the 23 studies provided age as a mean or median, and the reported minimum and maximum ages across all studies were 11 and 91 years, respectively [8,9,11,14,18,19,21,22,24,30,31,32,33,34,35]. Sex was reported in 12 of 23 studies; all 12 of 12 (100%) reported female-only cohorts [8,9,14,18,22,24,30,31,32,33,34,36], and sex was reported for only a subset of participants rather than the full study denominator in five of 12 studies [8,14,18,24,34]. Cohorts were predominantly lesion-/biopsy-based rather than strictly patient-based, consistent with an analytic focus on biopsy-targeted abnormalities and correlation outcomes.

### 3.1. Prevalence of Radiologic–Pathologic Discordance

Using the predefined review-level operational framework, discordance frequency remained heterogeneous and was therefore analyzed using stratified denominator frameworks, reflecting both source population selection and ascertainment strategy (Table 2). Discordant benign prevalence was directly estimable in 12 of 23 studies with an explicit denominator permitting calculation. In contrast, prevalence was not directly estimable in 11 of 23 studies because eligibility was restricted by design to discrepant-only cohorts or lesion-defined/excision-restricted populations [8,10,11,12,13,14,21,24,35,37,38]. Among studies using an inclusive all-biopsies denominator with cohort-wide radiologic–pathologic correlation, discordant benign prevalence ranged from 1.2% to 5.3% [9,19,30,31,32,33]. One study also used an all-biopsies denominator but restricted ascertainment of discordant benign lesions to cases proceeding to excision, yielding 7.4% (73/986); this proportion represented an excision-ascertained subset rather than a cohort-wide prevalence estimate [8]. When discordance was expressed using benign-biopsy denominators, reported proportions ranged from 1.5% to 19.2%, with the upper bound derived from an excision-restricted benign cohort [9,11,12,14,20,25,30,32,36]. In preselected high-suspicion strata where denominators were defined by BI-RADS severity and/or specific histologic categories, discordance was reported to be 1.5% (36/2385) in BI-RADS 4B–5 lesions with B1–B4 core needle biopsy histology [18]. However, discrepant-only or lesion-defined study designs did not permit estimation of prevalence in an unselected biopsy population [10,21]. Accordingly, direct comparison of prevalence estimates across studies should be interpreted cautiously because reported rates were dependent on denominator structure and eligibility design.

### 3.2. Clinical Outcomes of Cases with Radiologic–Pathologic Discordance

Malignancy upgrades among discordant benign lesions have been explicitly quantified in multiple studies (Table 2). The reported upgrade proportions ranged from 0% [12,31] to 100% in highly selected discordant subsets [11,32,37], reflecting important differences in study design, denominator selection, and case ascertainment. Across cohorts with broader clinical applicability, representative upgrade estimates commonly clustered between approximately 20% and 40%, with intermediate values including 26.0% (19/73) [8], 30.4% (7/23) [9], and 36.0% (9/25) [33], whereas several higher-risk cohorts reported rates exceeding 50%, such as 55.6% (20/36) [18]. Where histological subtypes were reported, malignant upgrades included both DCIS and invasive carcinoma. In the study by Çetin Tunçez et al., malignant upgrades among excised discordant benign lesions (*n* = 19) included DCIS in 11/19 (57.9%), invasive ductal carcinoma (IDC) in 4/19 (21.1%), encapsulated papillary carcinoma in 2/19 (10.5%), microinvasive DCIS in 1/19 (5.3%), and mucinous carcinoma in 1/19 (5.3%) [8]. In Vatteroni et al.’s study, malignancies detected at second-line ultrasound-guided VAB (US-VAB) (*n* = 17) comprised invasive carcinoma of no special type (NST) in 8/17 (47.1%), DCIS in 7/17 (41.2%), invasive lobular carcinoma (ILC) in 1/17 (5.9%), and angiosarcoma in 1/17 (5.9%), with additional malignant outcomes after non-upgrade at US-VAB, including DCIS in two cases at surgery and ILC in one case during surveillance, yielding an overall upgrade of 20/36 (55.6%) [18]. In a study by Poole et al., upgraded malignancies (*n* = 6) included invasive carcinoma in 2/6 (33.3%) and in situ carcinoma in 4/6 (66.7%) patients [24]. In a study by Lewin et al., upgraded cancers among discordant lesions (*n* = 9) comprised DCIS in 6/9 (66.7%) and IDC in 3/9 (33.3%) [33]. In the study by Son et al., upgraded malignancies among discordant lesions (*n* = 7) included IDC in 3/7 (42.9%), DCIS in 2/7 (28.6%), malignant phyllodes tumor in 1/7 (14.3%), and dermatofibrosarcoma in 1/7 (14.3%) [22].

### 3.3. Quality of Included Studies

Detailed domain-level quality assessments and overall appraisals of included studies are summarized in Table 3, Table 4 and Table 5 according to study design. Overall, most cohort studies were of moderate quality, with several higher-quality studies and one lower-quality study. Across these studies, the strongest domains were related to exposure ascertainment, outcome measurement, and statistical analysis, whereas confounding control and follow-up handling were the most frequent limitations. Confounding factors were unclear in 11 of 17 (65%) studies, explicit strategies to address confounding were absent in 7 of 17 (41%) studies, and only 1 of 17 (6%) studies clearly reported an adjustment strategy. Follow-up reporting and attrition management were also inconsistent, with incomplete follow-up in four of 17 (24%) studies and no stated approach to address incomplete follow-up in eight of 17 (47%) studies.

The risk of bias assessment for the studies appraised using the diagnostic test accuracy framework is presented in Table 4. Patient selection domains were generally acceptable (consecutive/random sampling: 4 of 5; avoidance of case–control designs: 5 of 5), whereas key concerns were concentrated in domains reflecting interpretive independence/blinding and analytic completeness. Interpretation of the index test without knowledge of the reference standard was unclear in 5 of 5 (100%) studies, and interpretation of the reference standard without knowledge of the index test was unclear in 4 of 5 (80%) studies and not met in 1 of 5 (20%) studies. Complete inclusion of patients in the analysis was not achieved in four of the five (80%) studies, indicating the potential for verification or attrition-related bias.

The analytical cross-sectional study met all eight JBI criteria and was therefore appraised as higher quality (Table 5), indicating clearly defined inclusion criteria, detailed participant and setting descriptions, valid and reliable exposure and outcome measurement, explicit consideration of confounding factors, and appropriate statistical analysis.

## 4. Discussion

This systematic review shows that radiologic–pathologic discordance after image-guided breast biopsy is generally uncommon at the population level, although it is clinically important because it concentrates residual risk among patients with missed or underestimated malignancy. In studies with cohort-wide correlation and an all-biopsy denominator, the discordant benign prevalence typically ranged from 1.2% to 5.3%, whereas an excision-ascertained design reported a prevalence of 7.4% (73/986), reflecting a selective verification rather than a true population prevalence. The most actionable phenotype was a discordant benign–benign (or otherwise non-explanatory) histology despite persistent imaging suspicion, as it signals potential sampling/targeting inadequacy and triggers escalation. Consistently, malignancy upgrade after additional tissue acquisition was frequently clinically meaningful across studies with quantifiable outcomes. Upgrade proportions ranged from 0% in some cohorts to more than 50% in highly selected populations, with several unselected or clinically relevant cohorts reporting rates around 20–40%. Therefore, even when discordant benign findings constitute a minority of biopsies, the nontrivial upgrade yield supports routine, structured radiology–pathology correlation, timely repeat biopsies (often vacuum-assisted, when appropriate), and surgical excision pathways to mitigate diagnostic delay.

### 4.1. Clinical Interpretation and Management Implications of Radiologic–Pathologic Discordance

Radiologic–pathologic discordance represents a failure of diagnostic reliability in image-guided biopsy, not merely an imaging–histologic discrepancy. Biopsy results are reliable only when they explain imaging findings, as disagreement suggests sampling errors and uncertain lesion status. Discordance indicates potential false-negative results or histological underestimation, particularly in malignant lesions [15,17]. Existing discordance rates vary because of differences in definitions, populations, techniques, and institutional practices. Structured multidisciplinary review improves concordance assessment and discordant case resolution [12,23]. Radiologic–pathologic concordance remains an important component of multidisciplinary breast care, where accurate tissue diagnosis supports appropriate downstream treatment planning and individualized management [39]. Radiologic–pathologic discordance should be considered a diagnostic safety event and quality metric in breast imaging, underscoring the importance of structured workflows, explicit concordance documentation, and multidisciplinary review to reduce diagnostic error, improve diagnostic accuracy, and support better clinical outcomes.

Interobserver variability in imaging interpretation, particularly within the BI-RADS framework, is an additional and often under-recognized contributor to variability in discordance. Although BI-RADS provides a standardized lexicon, interrater agreement is typically only moderate, with reported kappa values generally ranging from approximately 0.4 to 0.7, and lower agreement observed for descriptor assignment and intermediate assessment categories (e.g., BI-RADS 3–4) compared with clearly benign or highly suspicious lesions [40,41]. This variability has direct implications for discordance classification because discordance is inherently defined by the relationship between imaging-based suspicion and histological findings. Differences in the BI-RADS categorization may alter the thresholds for biopsy and influence whether a benign histological result is interpreted as concordant or discordant. Consequently, discordance reflects not only technical factors such as sampling adequacy but also the reproducibility of imaging interpretation, supporting the need for double reading, subspecialty expertise, and multidisciplinary review.

Discordant benign lesions represent the most clinically significant subgroup, which directly challenges the adequacy of percutaneous sampling when imaging suspicion persists. Malignancy upgrade rates following repeat biopsy or surgical excision range from 20 to 40%, reaching 50% in high-risk populations [8,9,18,19,33]. These rates exceed those of concordant benign findings, suggesting that discordant benign results are likely false-negative diagnoses. The upgraded lesions included both DCIS and invasive carcinoma, indicating that unresolved discordance may have caused stage migration. Image-guided breast biopsy techniques show false-negative rates of 1–3% for core needle biopsy and similar rates for VAB [42,43,44]. These rates may be higher in high-suspicion lesions because of targeting challenges or lesion heterogeneity. Studies have shown that missed cancers are typically identified through radiologic–pathologic discordance, which serves as a critical diagnostic safety mechanism [42]. Failure to recognize discordance may allow false-negative results to persist and delay diagnosis.

**Figure 2:**
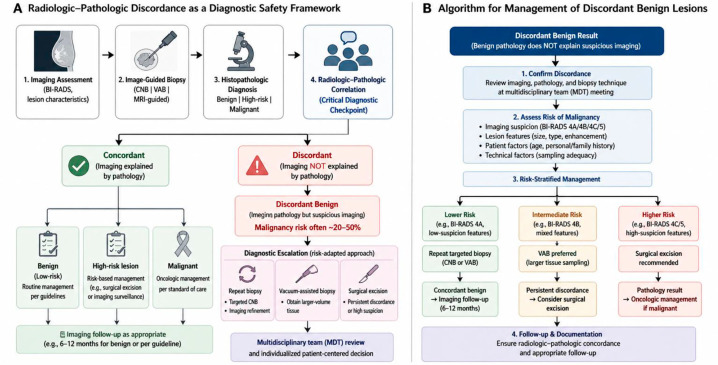
Radiologic–pathologic discordance as a diagnostic safety framework and algorithm for management of discordant benign lesions. (**A**) Radiologic–pathologic correlation is a critical diagnostic checkpoint after image-guided breast biopsy. Concordant results generally allow guideline-concordant management, whereas discordant results raise concern for a potential false-negative biopsy. Discordant benign lesions require diagnostic escalation with a risk-adapted approach, including repeat biopsy, vacuum-assisted biopsy (VAB), or surgical excision, based on multidisciplinary team (MDT) evaluation and patient-specific factors. (**B**) Algorithm for the management of discordant benign results, incorporating risk stratification, tissue sampling strategies, and follow-up to achieve diagnostic certainty while minimizing potential harm. Abbreviations: BI-RADS, Breast Imaging Reporting and Data System; CNB, core needle biopsy; VAB, vacuum-assisted biopsy; MRI, magnetic resonance imaging; MDT, multidisciplinary team.

These observations have direct implications for clinical management. The malignancy risk in discordant benign findings supports the diagnostic escalation of lesions with moderate to high imaging suspicion. Repeat tissue sampling via optimized biopsy or surgical excision remains essential because imaging surveillance alone is insufficient [9,15,33]. VAB may improve sampling adequacy, although false-negative results remain possible. Benign histologic results must be interpreted within the imaging context, with discordance prompting the reassessment of sampling adequacy. Clinical decisions should consider lesion characteristics and the histological context. Suspicious microcalcifications, architectural distortions, and higher BI-RADS categories warrant aggressive management because of the increased risk of malignancy. Nonspecific benign findings may not explain suspicious imaging features, whereas specific benign diagnoses that account for imaging findings support concordance. Concordance assessment requires the synthesis of imaging, pathological, and procedural factors. Radiologic–pathologic discordance represents a critical point, where failure to manage it appropriately may delay diagnosis. The risk of malignancy in discordant benign findings supports prompt reassessment and definitive diagnosis, reinforcing that benign histology should not override high imaging suspicion without proper correlation, as is demonstrated in Figure 2.

However, diagnostic escalation should be balanced against patient burden and potential harms. Across the included studies, harms were inconsistently reported, but several studies documented clinically relevant concerns, including patient refusal of recommended re-biopsy or excision, loss to follow-up [12,30,33], procedural pain or bleeding [18,36], hematoma after MRI-guided vacuum-assisted biopsy [32], higher costs or additional procedural burden [13,25], and potential overtreatment when excision ultimately yielded benign findings [21]. Therefore, repeat biopsy, VAB, surgical excision, or imaging follow-up should be selected through individualized radiologic–pathologic correlation, considering imaging suspicion, sampling adequacy, lesion type, patient comorbidity, local expertise, and patient preference.

### 4.2. Determinants and Predictors

Radiologic–pathologic discordance arises from various factors. It typically illustrates how pre-test imaging suspicion, sampling quality, and histologic diagnosis are connected to the targeted abnormality. In practice, higher suspicion assessments increase the chance of labeling a benign core result as discordant. For instance, BI-RADS 5 predicted true discordance in a recent large study of discordant benign lesions along with suspicious calcifications and architectural distortions [19]. Additionally, discordance was significantly associated with higher BI-RADS categories in the US-guided CNB group [45]. This aligns with the best-practice guidelines that consider benign results in highly suspicious lesions difficult to explain without clear supporting pathology [15,17].

Suspicious microcalcifications and architectural distortions independently predict true discordance [19]. Studies have shown lower interobserver agreement for distortions than for calcifications or spiculated masses [46]. Calcification morphology and distribution affect malignancy likelihood, raising standards for considering benign histology as “concordant” [15,16]. Lesion size has also been shown to be significant in univariate analysis but was not an independent predictor of true discordance [16,19]. In high-risk atypical ductal hyperplasia (ADH) cases, ultrasound lesions ≥ 15 mm can predict malignancy at excision [47]. Imaging visibility affects the modality choice and sampling confidence. For MRI-guided biopsies of additional lesions, patients with early-stage breast cancer have a higher cancer yield on the same side than on the opposite side. Laterality is crucial for probability prediction [48]. Age < 50 years has been associated with fewer surgical issues on the opposite side. Preoperative MRI “high-risk” features did not increase biopsy yield, which suggests that widespread MRI use as a prognostic tool may expose patients to low-yield targets [48]. Postoperatively, most new lesions were detected by US and MRI, whereas mammography identified fewer cases [49]. “Benign mimickers” can cause appearance–histology mismatches, increasing routine discordance [17,50,51].

Targeting accuracy and sampling adequacy are key procedural determinants across studies. Protocol elements that reduce the discordance risk include obtaining adequate cores, documenting post-fire needle position, and using calcification-specific precautions, such as specimen radiography and clip placement [15,16,17,19]. The biopsy guidance method has not been shown to be an independent predictor of true discordance [19], though variations in device size cause potential confusion. The tumor excision outcomes showed lower upgrade rates after VAB than after automated CNB. However, lesions that underwent repeat core biopsy before excision showed no upgrades, supporting repeat sampling when the initial adequacy was unclear [24]. In needle-localized biopsy, the wire distance to the target and specimen volume predict lesion removal failure [52]. For MRI-guided biopsy, discordance often results from MRI-targeting limitations such as, non-real-time imaging and contrast washout, with post-biopsy reviews revealing missed or partially sampled lesions [33,50].

Histological category influenced upgrade risk and concordance reliability. Borderline and high-risk entities, particularly ADH, had significant underestimation potential. In patients with ADH diagnosed using US-CNB, malignancy was detected during excision in 45.2% of patients. Older age and larger lesion size increase this risk [47]. In a series of discordant benign results, high-risk lesions appeared in “false discordance” cases, showing that benign outcomes can have clinical significance [19]. Studies have highlighted the differences between specific benign diagnoses and nonspecific findings, such as fibrocystic change and stromal fibrosis, which may be incidental and inadequate for suspicious imaging targets [15,17,50]. Adequate tissue sampling is crucial, with “insufficient sample with benign findings” associated with high upgrade rates [24]. Postoperative groups report “inadequate” benign categories, complicating correlations [49]. However, institutional correlation consistency affects discordance and multidisciplinary discussions may help to improve the correlation with breast conferences showing higher rates [46]. Clinical series have outlined the potential for management of discordant cases within multidisciplinary workflows [19,24,33]. This supports the need for direct radiologist-pathologist communication and structured reviews [50]. MRI-guided biopsies can delay surgery, particularly with poor results [48]. System gaps in documentation and communication reduce correlation benefits [46]. Some studies have reported limited application of radiopathology meetings [45]. Moreover, dedicated breast radiologist expertise and monitoring protocols can lead to lower discordance and reliable escalation decisions [33,49].

### 4.3. Methodological Heterogeneity and Risk of Bias Considerations

The interpretation of radiologic–pathologic discordance across studies is limited by substantial methodological heterogeneity, which affects both the estimated prevalence of discordance and the reported malignancy upgrade rates. A major source of variability is the lack of standardized definitions of discordance, ranging from subjective radiologist judgment to rule-based criteria (e.g., benign histology in BI-RADS 4–5 lesions), resulting in inconsistent classification and limited comparability across studies [15,17]. Additional heterogeneity arises from differences in denominators (all biopsies vs. benign biopsies), case mix (screening vs. high-suspicion populations), biopsy techniques, and imaging modalities, all of which influence the discordance prevalence and false-negative risk [9,33]. These factors likely contributed to the wide range of reported discordance rates and upgrade proportions observed in the literature. Denominator selection may also introduce interpretive bias, as estimates derived from benign-biopsy or excision-restricted cohorts can appear higher than cohort-wide all-biopsy estimates because they reflect selected higher-risk subgroups rather than true population prevalence.

Several clinically relevant sources of heterogeneity warrant further consideration. The imaging modality varied substantially across studies and included ultrasound-guided, stereotactic, tomosynthesis-guided, and MRI-guided pathways, each associated with different lesion spectra and sampling challenges. For example, MRI-guided cohorts more commonly involved MRI-only lesions or non-mass enhancement, whereas stereotactic cohorts frequently targeted calcifications or architectural distortion. Biopsy technique also differed across studies, including conventional core needle biopsy and vacuum-assisted biopsy using different needle gauges and sampling volumes, which may influence tissue adequacy and false-negative risk. In addition, lesion type and baseline suspicion level were inconsistent, with some studies restricted to BI-RADS 4B–5 lesions, stromal fibrosis, or lobular neoplasia. These differences likely contributed to variability in both discordance prevalence and reported malignancy upgrade rates.

Estimation of malignancy risk was further influenced by follow-up variability and verification bias. In many studies, only a subset of discordant lesions, typically those with higher imaging or clinical concerns, underwent repeat biopsy or surgical excision, potentially overestimating malignancy upgrade rates, whereas limited follow-up may underestimate false-negative diagnoses. Although image-guided breast biopsy demonstrates low overall false-negative rates (approximately 1–3%), missed cancers are frequently identified through radiologic–pathologic discordance, highlighting its role as a critical diagnostic safety mechanism [42,43,44]. Finally, the predominance of single-center observational studies, together with incomplete reporting of key variables, such as correlation criteria, sampling adequacy, and management pathways, introduces selection and reporting bias and limits generalizability. These limitations underscore the need for standardized definitions, uniform denominators, and core outcome sets, as well as stratified reporting by imaging modality, biopsy technique, and lesion type to improve comparability and support more robust risk estimation.

### 4.4. Limitations

This systematic review had several limitations that should be acknowledged. First, the included studies had substantial methodological heterogeneity in the definitions of radiologic–pathologic discordance, the denominators used for discordance rates, and concordance classification criteria. The lack of a universal definition limits the comparability across studies and prevents a quantitative synthesis. The reported estimates should be interpreted as context dependent rather than precise. Second, most of the included studies were retrospective, single-center observational studies, which are subject to selection bias, practice variability, and limited external validity, as differences in imaging expertise, biopsy techniques, and workflows may influence discordance identification and management. Third, malignancy risk estimation was affected by verification bias and incomplete follow-up. Many studies evaluated only a subset of discordant lesions with higher suspicion, potentially overestimating malignancy upgrade rates. Limited follow-up may result in the underdetection of false-negative results among concordant benign lesions. Inconsistent reporting of imaging characteristics, biopsy techniques, and correlation processes limited the ability to conduct a stratified analysis. Fourth, the discordance classification is subject to interobserver variability, particularly within the BI-RADS, where only moderate agreement exists between radiologists. This may affect both classification and management decisions. Publication bias should be considered, as English-language studies and clinically significant outcomes may have been overrepresented. Finally, most studies were conducted in North America, Europe, and selected Asian centers, with limited data from other regions and from male breast disease, which may also affect generalizability. Despite these limitations, the consistent finding of malignancy risk in discordant benign lesions supports the need for standardized definitions, prospective multicenter studies, and globally representative data.

## 5. Conclusions

This systematic review showed that radiologic–pathologic discordance after image-guided breast biopsy is uncommon but clinically consequential in unselected populations. In studies using an all-biopsy denominator, the prevalence of discordant benign lesions ranged from 1.2% to 5.3%. Among discordant benign lesions with additional sampling, malignancy upgrades ranged from 0% to 100% in selected subsets, reflecting substantial differences in study design and case selection. However, several clinically relevant cohorts reported more representative upgrade rates of approximately 20–40%, while higher-risk subsets reached 50% or more. Both ductal carcinoma in situ and invasive carcinoma were identified on subsequent tissue assessment. These findings support a systematic radiologic–pathologic correlation and timely diagnostic resolution with repeat sampling (including vacuum-assisted techniques when appropriate) and/or surgical excision rather than surveillance alone. However, interpretation is limited by the heterogeneous definitions, nonuniform denominators, and potential confounding and the handling of patient follow-up. Future prospective multicenter studies with standardized criteria, uniform denominators for all biopsied lesions, and consistent outcome reporting are needed to refine prevalence estimates and management strategies.

## Figures and Tables

**Table 1:** Characteristics of Included Studies.

Study	Setting/Design/Period	Population/Lesions	Imaging Context	Biopsy Technique (Key Parameters)	Radiologic–Pathologic Correlation/Discordance Handling
[34]	Jordan; tertiary hospital; retrospective single-center study; 2015–2022	BI-RADS IVc/V lesions undergoing biopsy; 828 lesions. Benign initial biopsy 44/828; discordant benign 26/828. All discordant analytic cases were female (mean age 52.7 years, range 26–91)	Ultrasound-based BI-RADS IVc/V classification. Among benign-at-biopsy cases: BI-RADS IVc 34/44 and BI-RADS V 10/44. Lesion types included masses and calcifications	Predominantly US-guided CNB; stereotactic VAB in a minority (40/44 vs. 4/44 among benign-at-biopsy cases). Needle gauge not reported	Discordance conceptually defined as BI-RADS IVc/V with benign pathology requiring clinicoradiologic–pathologic correlation and MDT review. Repeat biopsy in 20/44 benign-at-biopsy cases; 10 malignant and 10 benign. Sixteen of 44 classified benign after MDT review
[8]	Turkey; Izmir City Hospital; retrospective; 2020–2023	Patients undergoing breast CNB. Analytic discordant cohort: 73 women with BI-RADS 4–5 lesions and non-malignant CNB proceeding to excision/lumpectomy	Final BI-RADS assigned by US (*n* = 5), mammography (*n* = 19), or MRI (*n* = 49). BI-RADS distribution: 4A 17, 4B 38, 4C 16, 5 2	US-guided CNB only; 18G needle via 17G coaxial system; ≥3 specimens	Discordance defined as persistent clinical/imaging suspicion not explained by CNB. Excision recommended after multidisciplinary review; non-excised benign CNB cases underwent 2-year imaging follow-up
[18]	Italy; single-center retrospective study; September 2020–April 2023	Consecutive women undergoing US-guided CNB for BI-RADS 4–5 lesions; 2385 lesions. Discordant cohort: 36 lesions	Ultrasound BI-RADS (5th edition). Discordant lesions: BI-RADS 4B 16, 4C 18, 5 2	First-line US-guided 14G CNB (≥3 cores). Second-line US-guided 10G VAB (≥6 cores) with clip placement	Concordance assessed by radiologist with breast pathologist consensus. Discordance defined as BI-RADS 4B/4C/5 with B1–B4 histology. All discordant lesions underwent second-line VAB; surgery or follow-up used as reference standard
[19]	Hong Kong SAR, China; Queen Elizabeth Hospital; retrospective database study; 2012–2021	3080 breast biopsies; 64 discordant benign lesions (2.1%). Additional workup available for 55 lesions	Mammography plus ultrasound interpreted using BI-RADS. Suspicious lesions BI-RADS 4–5	US-guided CNB (14G; minimum 3 cores) or stereotactic 9G VAB. Clip placement for calcification targets	Discordance established in multidisciplinary meetings. True discordance = malignancy on repeat biopsy/excision; false discordance = benign confirmation
[30]	South Africa; Charlotte Maxeke Johannesburg Academic Hospital; retrospective review; August 2016–July 2019	131 stereotactic biopsies from 123 women	Mammographically detected, sonographically occult abnormalities	Prone stereotactic or DBT-guided biopsy. Predominantly 9G VAB (129/131); limited CNB (2/131)	Discordance identified when imaging findings were not explained by pathology. Additional tissue sampling and/or surgery performed
[12]	USA; community teaching hospital; retrospective review of conference database and records; 2013–2018	Selected benign image-guided CNB conference cases; 393 cases	BI-RADS used pre-biopsy; clinically/radiologically challenging benign cases	Image-guided CNB under US, stereotactic, or MRI guidance; device details not reported	Monthly multidisciplinary conference determined concordance vs. discordance and recommended repeat biopsy, excision, imaging review, or follow-up
[31]	USA; single institution; retrospective; June 2017–January 2020	DBT-detected architectural distortion without sonographic correlate; 151 lesions	DBT lesion with negative targeted US	DBT-guided 9G VAB; median 7 cores	Discordance assessed by performing radiologist; discordant benign lesions underwent surgical excision
[10]	Switzerland; University Hospital Zurich and affiliated centers; retrospective; 2010–2019	Discrepant preoperative biopsy cases only; 232 patients	Primarily ultrasound BI-RADS assessment	Initial biopsy always US-guided CNB; later increasing re-biopsy/VAB use	Discordant cases underwent further imaging correlation, second-look imaging ± MRI, then re-biopsy and/or surgery via interdisciplinary decision-making
[11]	USA; Emory University School of Medicine; retrospective; December 2009–July 2017	390 stereotactic biopsies for calcifications; analytic LN cohort 81 biopsies	Mammographically detected suspicious calcifications	Stereotactic VAB (9G or 11G); mean 11 cores	Dedicated breast radiologist assigned concordance/discordance; discordant lesions proceeded to excision
[21]	USA; Mount Sinai Dubin Breast Center; retrospective; 2001–2014	Pure LN on CNB; 178 patients/cases	Microcalcifications 80%, mass 20%; mammography, US, or MRI	Image-guided CNB; gauge not reported	Mandatory radiologist–pathologist correlation. Discordance when histology did not explain imaging abnormality
[33]	USA; New York University School of Medicine; retrospective; October 2007–October 2013	MRI-guided VAB cohort; 1314 lesions in 1211 women	MRI-detected masses or non-mass enhancement	MRI-guided 9G VAB; standard 10–12 samples; marker placement	Concordance determined by biopsying radiologist. Surgical excision recommended for all discordant lesions
[32]	France; two cancer referral centers; retrospective observational study; January 2009–February 2013	208 MRI-biopsied lesions in 197 women	MRI-visible lesions, mainly BI-RADS 4–5	MRI-guided VAB using 10G or 11G systems; median 18 cores	Correlation included clip position and MRI features. Discordant lesions proceeded to surgical excision; concordant benign lesions had MRI follow-up
[9]	USA; single institution; retrospective; January 2005–December 2012	1861 stereotactic VAB lesions	Predominantly calcifications (76%)	9G or 11G stereotactic VAB; 10–12 specimens routinely	Discordance documented by radiologist after pathology review. All discordant lesions recommended for surgical excision
[24]	USA; Los Angeles County Medical Center and USC Norris Center; retrospective; January 2008–June 2013	81 patients undergoing excision for benign discordant BI-RADS 4–5 lesions	BI-RADS 4A–5 lesions; multiple imaging guidance routes	Prior CNB or VAB using multiple gauges/devices	Multidisciplinary review determined discordance. Cohort restricted to lesions proceeding to excisional biopsy
[37]	Canada; The Ottawa Hospital; retrospective chart review; January 2005–December 2009	Stromal fibrosis cohort; 365 lesions	Only BI-RADS 4–5 lesions biopsied	CNB (14G/16G) or VAB (10–12G); predefined minimum cores	Radiology–pathology correlation performed. Repeat biopsy or surgery recommended for discordant cases
[38]	Republic of Korea; Samsung Medical Center; retrospective; January 2007–December 2008	Stromal fibrosis cohort; 187 lesions in 181 patients	Mammography and sonography; BI-RADS 2–5	US-guided 14G CNB, US-guided 11G VAB, or stereotactic 11G VAB	Correlation performed for all lesions. Discordant lesions underwent immediate re-biopsy
[25]	USA; Bryn Mawr Hospital Breast Center; retrospective; January–December 2010	689 patients undergoing image-guided biopsy	Image-detected abnormalities with BI-RADS follow-up pathways	Stereotactic 9G VAB, US-guided 12G VAB or 14G CNB, MRI-guided 9G VAB	Radiologist addendum classified concordance vs. discordance. Most discordant lesions underwent excision
[35]	USA; Mayo Clinic; retrospective cohort; January 1993–December 2010	Pure LN on CNB; 184 cases from 180 patients	Calcifications 74%, mass/nodule 21%, MRI enhancement 5%	Stereotactic, US-guided, or MRI-guided CNB; gauges 9G–18G	Dedicated radiology and pathology re-review performed. Discordant lesions usually recommended for excision
[22]	South Korea; Yonsei University and Kangbuk Samsung Hospital; prospective observational; January 2005–December 2006	Benign CNB cohort with verification; 1588 lesions in 1444 women	Ultrasound-visible lesions using US BI-RADS	US-guided 14G CNB; standard 5–6 cores	Prospective radiology–pathology review within 1 week. Discordance assigned when benign histology did not explain suspicious imaging
[14]	South Korea; Yonsei University College of Medicine; retrospective; February 2000–June 2005	2985 BI-RADS 4–5 lesions biopsied; discordant analytic cohort 74 lesions	Sonographically guided biopsy of BI-RADS 4–5 lesions	Freehand US-guided 14G CNB; typically 5–6 cores	Two radiologists reviewed imaging and pathology. Discordant lesions proceeded to excision
[20]	Israel; Soroka University Medical Center; consecutive series; October 1998–September 2001	Solid breast lesions requiring diagnosis; 715 lesions in 652 patients	Ultrasound-visible lesions	US-guided 14G CNB; median 4 cores	Imaging–histology correlation performed. Repeat biopsy recommended for disagreement; benign cases had structured follow-up
[36]	USA; multicenter prospective consortium trial (22 sites); mid-1990s	Nonpalpable mammographic lesions; analytic cohort 1681 lesions	Mammographically detected masses or calcifications	Stereotactic or US-guided large-core biopsy; minimum ≥ 5 cores	Standardized radiology–pathology workflow. Surgical excision mandated for discordance or high-risk diagnoses
[13]	Austria; University of Vienna Medical School; retrospective validation study; September 1997–December 2001	Consecutive stereotactic VAB lesions with subsequent excision; 318 lesions	Mammographic lesions including calcifications, masses, asymmetry, distortion	Stereotactic 11G VAB; average 15–20 specimens	Discordance defined when histology did not explain imaging findings. All lesions underwent surgical excision per institutional protocol

Note. BI-RADS, Breast Imaging Reporting and Data System; CNB, core needle biopsy; DBT, digital breast tomosynthesis; LN, lobular neoplasia; MDT, multidisciplinary team; MRI, magnetic resonance imaging; US, ultrasound; VAB, vacuum-assisted biopsy.

**Table 2:** Prevalence and Clinical Outcomes of Radiologic–Pathologic Discordance.

Study	N	Denominator Type	Discordant Benign Prevalence	Management	Upgrade
[34]	828 lesions	High-suspicion subgroup	26/828 (3.1%)	MDT review; repeat biopsy; excision	10/20 (50.0%)
[8]	986 CNB patients	Excision-restricted cohort	73/986 (7.4%)	Excision	19/73 (26.0%)
[18]	2385 lesions	High-suspicion subgroup	36/2385 (1.5%)	VAB; excision; follow-up	20/36 (55.6%)
[19]	3080 biopsies	All biopsies	64/3080 (2.1%)	Repeat biopsy; excision	17/55 (30.9%)
[30]	131 biopsies	All biopsies	4/131 (3.1%)	Repeat biopsy; excision	2/4 (50.0%)
[12]	393 cases	Benign biopsies only	22/393 (5.6%)	Repeat biopsy; excision; follow-up	0/8 (0.0%)
[31]	151 biopsies	Benign biopsies only	8/151 (5.3%)	Excision	0/8 (0.0%)
[10]	232 patients	High-suspicion subgroup	Not directly estimable	Repeat biopsy; excision; follow-up	26/57 (45.6%)
[11]	81 biopsies	Lesion-defined selected cohort	Not directly estimable	Excision	1/1 (100.0%)
[21]	178 patients	Lesion-defined selected cohort	Not directly estimable	Excision; follow-up	8 cases reported
[33]	1314 biopsies	All biopsies	25/1314 (1.9%)	Excision; follow-up	9/25 (36.0%)
[32]	208 lesions	Benign biopsies only	4/147 (2.7%)	Excision	4/4 (100.0%)
[9]	1861 biopsies	All biopsies	23/1861 (1.2%)	Excision	7/23 (30.4%)
[24]	81 patients	Excision-restricted cohort	Not directly estimable	Excision	6/81 (7.4%)
[37]	365 patients	Lesion-defined selected cohort	16/365 (4.4%)	Repeat biopsy; excision	16/16 (100.0%)
[38]	187 lesions	Lesion-defined selected cohort	6/187 (3.2%)	Repeat biopsy	3/6 (50.0%)
[25]	689 patients	Benign biopsies only	11/498 (2.2%)	Repeat biopsy; excision; follow-up	3/11 (27.3%)
[35]	184 patients	Lesion-defined selected cohort	13/184 (7.1%)	Excision; follow-up	1/10 (10.0%)
[22]	1588 lesions	Benign biopsies only	103/1588 (6.5%)	Repeat biopsy; VAB; excision; follow-up	7/103 (6.8%)
[14]	2985 lesions	Excision-restricted cohort	74/386 (19.2%)	Excision	13/74 (17.6%)
[20]	715 lesions	Benign biopsies only	14/399 (3.5%)	Repeat biopsy; excision	7/14 (50.0%)
[36]	1681 patients	Benign biopsies only	119/1214 (9.8%)	Repeat biopsy; excision	9/119 (7.6%)
[13]	318 lesions	Excision-restricted cohort	13/318 (4.1%)	Excision	7/13 (53.8%)

Note. Data are presented as n/N (%) where available. Discordant benign prevalence should be interpreted according to the reported denominator type. Selected cohorts may not represent cohort-wide prevalence estimates. Not directly estimable indicates that prevalence could not be calculated because the study population was restricted by design. CNB, core needle biopsy; MDT, multidisciplinary team; VAB, vacuum-assisted biopsy.

**Table 3:** Quality Assessment of Cohort Studies (JBI Checklist).

Study	Q1	Q2	Q3	Q4	Q5	Q6	Q7	Q8	Q9	Q10	Q11	Overall
[8]	Y	U	Y	U	U	Y	Y	NA	U	NA	Y	Moderate
[19]	Y	Y	Y	U	Y	Y	Y	N	N	N	Y	Moderate
[30]	NA	NA	Y	NA	NA	NA	Y	N	Y	N	Y	Moderate
[12]	NA	NA	Y	U	U	Y	Y	U	N	N	Y	Moderate
[31]	NA	NA	Y	NA	NA	NA	Y	NA	NA	NA	Y	Moderate
[10]	U	Y	Y	U	U	Y	Y	U	U	N	Y	Moderate
[11]	Y	Y	Y	U	N	Y	Y	Y	U	N	Y	Moderate
[21]	Y	Y	Y	U	N	Y	Y	Y	N	N	Y	Moderate
[33]	NA	NA	Y	NA	NA	Y	Y	Y	Y	NA	Y	Higher quality
[32]	NA	NA	Y	U	N	NA	Y	Y	Y	NA	Y	Moderate
[9]	NA	NA	Y	U	N	U	Y	NA	Y	NA	Y	Moderate
[24]	NA	NA	Y	NA	NA	Y	Y	NA	NA	NA	Y	Higher quality
[37]	NA	NA	Y	NA	NA	Y	Y	Y	U	U	Y	Higher quality
[25]	U	U	U	N	N	Y	Y	U	N	N	Y	Lower quality
[35]	Y	Y	Y	U	N	Y	Y	Y	U	N	Y	Moderate
[22]	Y	Y	Y	U	U	Y	Y	Y	Y	NA	Y	Higher quality
[14]	NA	NA	Y	U	N	U	Y	Y	Y	NA	Y	Moderate

Note. JBI, Joanna Briggs Institute; Q1–Q11, items of the JBI Critical Appraisal Checklist for Cohort Studies: Q1, similarity of groups and recruitment from the same population; Q2, exposure measured similarly in groups; Q3, exposure measured in a valid and reliable way; Q4, confounding factors identified; Q5, strategies to deal with confounding stated; Q6, participants free of the outcome at baseline; Q7, outcomes measured in a valid and reliable way; Q8, follow-up time reported and sufficient; Q9, follow-up complete (or losses described and explored); Q10, strategies to address incomplete follow-up; Q11, appropriate statistical analysis. Y, yes; N, no; U, unclear; NA, not applicable.

**Table 4:** Quality Assessment of Diagnostic Test Accuracy (JBI Checklist).

Study	Q1	Q2	Q3	Q4	Q5	Q6	Q7	Q8	Q9	Q10	Overall
[18]	Y	Y	U	U	NA	U	U	Y	N	Y	Moderate
[38]	Y	Y	U	U	NA	U	U	N	N	Y	Moderate
[20]	Y	Y	Y	U	NA	Y	N	Y	N	Y	Moderate
[36]	U	Y	U	U	NA	Y	U	N	N	Y	Lower quality
[13]	Y	Y	N	U	NA	Y	U	U	Y	N	Moderate

Note. JBI, Joanna Briggs Institute; Q1–Q10, items of the JBI Critical Appraisal Checklist for Diagnostic Test Accuracy Studies: Q1, patient selection was consecutive or random; Q2, a case–control design was avoided; Q3, inappropriate exclusions were avoided; Q4, index test results were interpreted without knowledge of the reference standard results; Q5, prespecified threshold was used; Q6, reference standard likely correctly classified the target condition; Q7, reference standard results were interpreted without knowledge of the index test results; Q8, appropriate interval between index test and reference standard (and all patients received the same reference standard); Q9, all patients were included in the analysis; Q10, withdrawals/indeterminate results were reported and handled appropriately. Y, yes; N, no; U, unclear; NA, not applicable.

**Table 5:** Quality Assessment of Analytical Cross-Sectional Studies (JBI Checklist).

Study	Q1	Q2	Q3	Q4	Q5	Q6	Q7	Q8	Overall
[34]	Y	Y	Y	Y	Y	Y	Y	Y	Higher quality

Note. JBI, Joanna Briggs Institute; Q1–Q8, items of the JBI Critical Appraisal Checklist for Analytical Cross-Sectional Studies: Q1, criteria for inclusion in the sample were clearly defined; Q2, study subjects and the setting were described in detail; Q3, exposure was measured in a valid and reliable way; Q4, objective, standard criteria were used for measurement of the condition; Q5, confounding factors were identified; Q6, strategies to deal with confounding factors were stated; Q7, outcomes were measured in a valid and reliable way; Q8, appropriate statistical analysis was used. Y, yes.

## Data Availability

All data supporting the findings of this study are included within the manuscript. Additional details or clarifications can be provided upon reasonable request to the corresponding author.

## Supplementary Materials

The following supporting information can be downloaded at: https://www.mdpi.com/article/10.3390/medsci14020229/s1, File S1: PRISMA 2020 checklist. File S2: Reproducible extracted dataset of study characteristics, biopsy variables, radiologic–pathologic discordance, management, and malignancy upgrade outcomes.

## Abbreviations

The following abbreviations are used in this manuscript:

ADH	Atypical ductal hyperplasia
BI-RADS	Breast Imaging Reporting and Data System
CNB	Core needle biopsy
DCIS	Ductal carcinoma in situ
DOAJ	Directory of Open Access Journals
IDC	Invasive ductal carcinoma
ILC	Invasive lobular carcinoma
INPLASY	International Platform of Registered Systematic Review and Meta-Analysis Protocols
JBI	Joanna Briggs Institute
MEDLINE	Medical Literature Analysis and Retrieval System Online
MRI	Magnetic resonance imaging
NST	No special type
PRISMA	Preferred Reporting Items for Systematic Reviews and Meta-Analyses
US	Ultrasound
US-VAB	Ultrasound-guided vacuum-assisted biopsy
VAB	Vacuum-assisted biopsy
